# Open questions: Reflections on plant development and genetics

**DOI:** 10.1186/1741-7007-11-25

**Published:** 2013-03-28

**Authors:** Virginia Walbot

**Affiliations:** 1 Department of Biology, Stanford University, Stanford, CA 94305, USA

## Genomics has given us a new appreciation for the many ways genes and genomes evolve

At the turn of the millennium, the most we could hope for was a few small genomes completed and hordes of ESTs and genomic snippets from most species. What a difference a decade makes. Now that whole genome sequencing is routine and there are sufficient numbers of plant genomes with near complete assemblies with strong support, it will be routine to ‘paste on’ the genomes of related genera and even families. Furthermore, using the multiple (hundreds or thousands) of distinct genomes of inbred lines or ecotypes, allelic diversity and micro-heterogeneity in chromosome organization can be analyzed directly. These assemblages can be used to test ideas about genome rearrangement, the timing and extent of transposon amplification, gene duplications and losses, and both promoter region and transcription unit conservation and change. But has genomics explained development or physiology?

## Genetics still has a role because mechanistic insights emerge from detailed analysis of a particular organism and with genomics we can tackle very difficult phenotypes

Despite the success of genomics, biological details are best viewed by close observation and analysis of a single species. In the context of all genes in that organism, what is the impact of mutation in one gene? What are the details of biochemical and gene expression regulation in the cells of this particular plant under specific environmental conditions? In the past 10 years, genomics has given us the ability to observe and dissect the small accretions in phenotype typical of multi-locus traits. We have unprecedented access to analysis of quantitative traits, that is, how a few or even dozens of specific allelic combinations at many loci add up to a particular trait such as days to flowering or ability to resist salt damage. Of course, classical genetics found the major players - lethal mutations always prove that something is essential - but the small number of major players do not explain the endless variety of intermediate types and just slightly more somatically and reproductively fit individuals under specific conditions.

## Real plants live in a variable environment and integrate environmental conditions into developmental decisions

As a corn geneticist, I’ve always faced the variability of growing conditions - day length, temperature, winter Hawaii compared to summer California, and so on. We observe phenotypic plasticity all the time, and now we are joined by those studying ‘growth chamber’ species who have begun to aggressively and effectively assess phenotypes in natural environments and diverse ecotypes in common gardens. The unprecedented combination of genome markers and high-throughput phenotyping is inspiring a new generation of ecophysiologists. It has been nearly 75 years since Clausen, Keck and Hiesey of Stanford/Carnegie Institution began publishing their common garden experiments that established that ecotypes differ genetically and that plants can show significant acclimations in form.

This latter point was noted by Charles Darwin, with particular reference to reproduction in his book *The Different Forms of Flowers on Plants of the Same Species *[1]. Typical open pollinated species that suffer low seed set can subsequently switch to making cleistogamous (closed bud), self-fertile flowers to ensure reproduction. It is endlessly fascinating to me how well plants cope with a variable environment and can, for example, produce a succession of leaves of different phenotypes to avoid sun, wind or other damage. Although the common wisdom is that physiology feeds into development by fine-tuning organ growth, our own work points to a deeper connection in that hypoxia is the regulator of the differentiation of maize anther cells competent for meiosis [2] and provides an example of a direct connection between cell fate setting and environmental conditions.

## The meaning of stem cells and the fundamental differences between plants and animals

Since the 1950s it has been clear that some individual adult plant cells can regenerate an entire organism. This was thought impossible in multicellular animals, but in the past few years it has become clear that animal stem cells can be reprogrammed *in vitro* to exhibit pluripotency or totipotency. But this is not easy! Why can plant cells dedifferentiate and redifferentiate autonomously, without a somatic niche helper cell population or an onslaught of special factors applied exogenously? One could argue that even a large tree is just cells that are currently cooperating to make a larger organism but that most of the cells retain a somewhat ‘single cell’ perspective on survival. The absence of a plant germ line may be the fundamental feature that divides plants from animals and may in ways we will ultimately understand determine the plasticity of plant cells within a complex multicellular organism. There is no doubt that a typical animal stem cell is so much more limited in what it can do, or does do in the body, than a shoot apical meristem cell from a flowering plant. In effect the plant stem cells are building entire new organs all the time - new limbs, new trunk - solving polarity issues and the other key developmental decisions that are resolved in animal embryos. Furthermore, in the very act of generating a new leaf, the shoot apex not only regenerates itself but it makes an axillary meristem, doubling the growth potential of the organism.

A favorite thought of mine is that this vegetative diversification of growing points - a kind of distributive growth - permits plants the luxury of mutation and an immediate assessment of fitness vegetatively. Animals sequester their germ line and stem cells to prevent mutation while plants may allow, even promote through activation of transposons, genome mutation. Novelty such as bud sports is the foundation for viticulture and tree crop diversification, evidence that some somatic mutations are highly favorable. And then when apices switch to making flowers the more successful branches will make more flowers and hence have the potential to make more offspring inheriting a ‘pre-tested’ allele that confers novel somatic properties. As a bonus, with gametophytic selection acting on the haploid phase of the life cycle, many highly deleterious mutations are eliminated from the plant gene pool, curtailing an increase in genetic load.

In summary, the past decade has provided many exciting scientific advances and a few solutions to long-standing questions. Yet the frontier of unanswered questions is still vast, and the challenge is to marshal our new resources to design appropriate and clever (and in these times, economical) approaches to resolving the mechanisms underlying the fundamental properties of the green world.
